# Combination treatment with nivolumab and Rigvir of a progressive stage IIC skin melanoma patient

**DOI:** 10.1002/ccr3.2182

**Published:** 2019-05-08

**Authors:** Linda Brokāne, Inta Jaunalksne, Andra Tilgase, Evija Olmane, Donatas Petroška, Agnija Rasa, Pēteris Alberts

**Affiliations:** ^1^ AmberLife Cancer Clinic Jūrmala Latvia; ^2^ Rigvir Rīga Latvia; ^3^ Department of Radiology Pauls Stradiņš Clinical University Hospital Rīga Latvia; ^4^ National Center of Pathology Affiliate of Vilnius University Hospital Santaros Klinikos Vilnius Lithuania

**Keywords:** combination treatment, metastatic melanoma, nivolumab, oncolytic virus, Rigvir

## Abstract

A 35‐year‐old male patient was diagnosed with stage IIC skin melanoma that rapidly progressed after surgery. Treatment was continued with radiotherapy, which did not stop further spread of disease and the patient was put on a combination of nivolumab and Rigvir. Subsequently, the progression has slowed.

## 1 INTRODUCTION

The treatment of melanoma has changed significantly over the last decade. Three classes of new treatment approaches—BRAF inhibitors, immune checkpoint therapy (CTLA‐4 and PD‐1 antibodies), and oncolytic viruses, have been developed, and several new agents have been approved.^1^

Nivolumab is one of the monoclonal anti‐PD‐1 antibody treatments. Nivolumab binds to the PD‐1 ligand, which is expressed on tumor cells. Normally, the PD‐1 receptor is expressed on B and T cells, as well as on monocytes and natural killer cells. Tumor cells express the PD‐1 receptor ligand that inhibits the proliferation and survival of CD8^+^ cytotoxic cells.^2^ Nivolumab and another anti‐PD‐1 agent, pembrolizumab, have recently been registered by FDA as treatment for metastatic melanoma. The results of clinical trials showed increased response rates and overall survival in comparison with standard chemotherapy.^3^, ^4^

Although significant progress has been made, current immune checkpoint therapies cause severe adverse events and better overall survival is still needed.^5^ To improve overall survival in patients, several new clinical trials focus on combination therapies. Mainly, immune checkpoint therapies are combined with other therapies such as oncolytic viruses.^1^

Rigvir is an oncolytic ECHO‐7 virus strain belonging to the *Picornaviridae* family, Enterovirus genus. Rigvir is a positive sense single‐stranded RNA virus selected and adapted for melanoma.^6^ In a postmarketing retrospective study, stage IB and stage II melanoma patients that had received Rigvir therapy had 4.39‐ to 6.57‐fold lower mortality than the observation group.^7^

Here, we report a case where a metastatic melanoma patient has been treated with the oncolytic virus Rigvir and the PD‐1 monoclonal antibody nivolumab.

## 2 CASE DESCRIPTION

**Figure 1:**
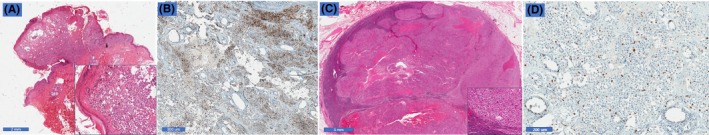
(A) Histological microphotographs. Skin invasive ulcerated nodular melanoma, invasion III level by Clark. Hematoxylin and eosin stain (x25; inclusion HE x 400). Scale bar is 2 mm. (B) Skin invasive nodular melanoma. Positive HMB‐45 antibody stain (x150). Scale bar is 300 μm. (C) Melanoma metastasis (19 mm) in lymph node, at 3.7 mo. Hematoxylin and eosin stain (x20; inclusion HE x 400). Scale bar is 3 mm. (D) Skin invasive nodular melanoma. Ki67 antibody stain (x20). Scale bar is 200 μm

A male patient at the age of 35 noticed a mole on his right shoulder and underwent surgical removal of the mole. Histological examination suggested stage IIC nodular melanoma (pT4bN0M0), invasion level by Clark III, Breslow's depth 4.5 mm with ulceration, and excision with tumor distance of 2.2 mm from lateral surgical margin ((Figure 1A), HMB‐45 positive (Figure 1B)). Up to three mitoses per 1 mm^2^ were observed in dermal component of the tumor. The pathological examination of the sentinel node was negative. However, after a month the patient felt a growth in the area of right shoulder blade. After a month, a scar widening and removal of three sentinel lymph nodes were performed. All nodes were without malignant changes. At 2.2 months, metastases in the right collar bone and under the right shoulder (classified as nodular and local recurrence) were detected. The tumor was negative for BRAF V600E mutation. At 3.2 months, new metastases above the right collar bone and under the right shoulder appeared, and eight lymph nodes above the right collar bone were removed; the histopathology report confirmed melanoma metastasis (of 19 mm diameter) in one lymph node (Figure 1C). Immunohistochemically, primary tumor and metastases excised at 2.2 months showed moderate/strong cytoplasmic staining in 50% of atypical cells for HMB‐45, moderate/strong cytoplasmic staining in 95% of atypical cells for MelanA, and strong cytoplasmic and nuclear staining in 100% of atypical cells for S100. Ki67 proliferation index was not more than 20% (Figure 1D).

Between 4.7 and 5.3 months, the patient received radiotherapy to the right axilla, right shoulder blade, and right clavicula (9 × 4 Gy, 36 Gy in total).

**Figure 2:**
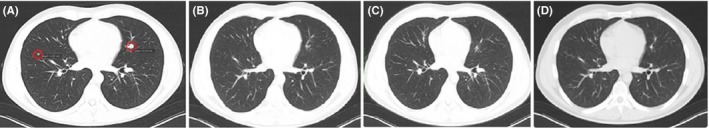
Contrast‐enhanced CT scans at (A) 6.6 mo show several lung nodules, with largest nodule in the lingular lobe of the left lung. Lung nodules and enlarged lymph nodes in mediastinum are not seen at (B) 10.2 mo, (C) at 1.3 y, and (D) at 1.5 y showed no change in size

At 5 months, excision of one node from the right armpit was performed and the whole node was full of melanoma metastasis. A CT chest scan at 6.6 months detected a neoplastic process spreading to the right lobe of the lung and mediastinum on both sides, and two large lumps in the right lung suggested metastases (Figure 2A, 3A‐C). Pathological lymph nodes in the left side of mesentery were detected at 7 months (Figure 4A). Hypovascular focal lesions in the right liver lobe and spleen, enlarged lymph node in hepatic hilus, were also observed (Figure 5A,B).

**Figure 3:**
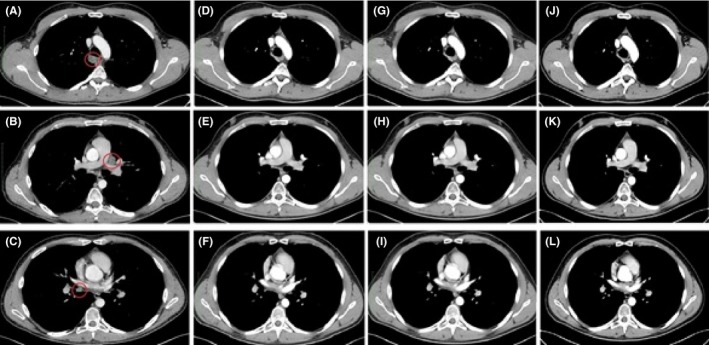
(A, B, C) Contrast‐enhanced CT scans reveal enlarged lymph nodes in the mediastinum with the largest diameter of 1.4 cm at 6.6 mo. CT scans show no enlarged lymph nodes in the mediastinum at (D, E, F) 10.2 mo, (G, H, I) 1.3 y, and (J, K, L) 1.5 y

**Figure 4:**
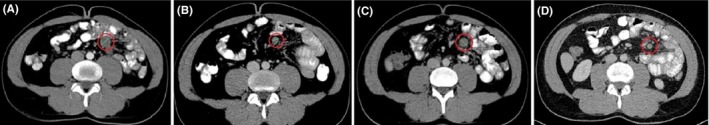
Contrast‐enhanced CT scans reveal pathological lymph nodes in the left side of the mesentery with the largest lymph node of up to 2 cm in the largest diameter at (A) 7 mo that has decreased in size to 1 cm in the largest diameter at (B) 10.2 mo. (C) Decrease in size was observed from 1.3 y when the lymph node had increased to 1.6 cm. At (D) 1.5 y, the size of the lymph node has decreased to 0.9 cm

**Figure 5:**
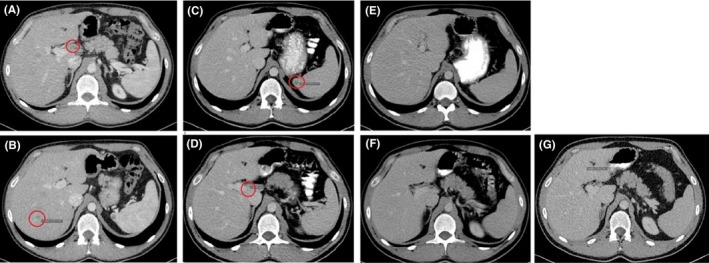
Abdominal contrast‐enhanced CT late phase scans at 6.6 mo show (A) a hypovascular focal lesion in the right lobe of the liver of up to 1 cm in diameter and (B) in the spleen up to 1 cm. (C, D) In the scan at 10.2 mo, the lesion in right liver lobe of the liver had disappeared and the lymph node in the hepatic hilus has been reduced in size. (E, F) At 1.3 y and (G) at 1.5 y, there is no change in size of the lymph nodes and lesions in the liver are not observed

**Figure 6:**
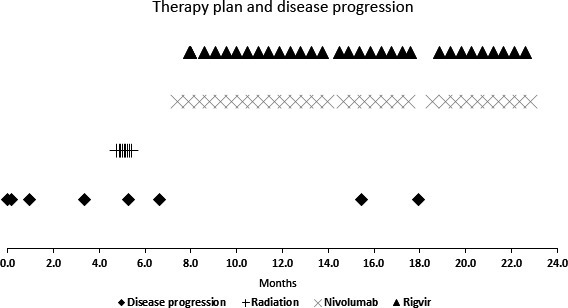
Therapy plan and disease progression dates

At 7.2 months, according to CT scans (Figures 4A and 5A,B) disease progression to the liver, spleen, and abdominal lymph nodes was once again suspected. At 7.3 months, the patient started immunotherapy with nivolumab 3 mg/kg (240 mg) iv infusions (2‐week intervals), and at 7.8 months—patient started virotherapy with Rigvir 2 ml (im also 2‐week intervals on alternating weeks). The patient has received 32 injections of Rigvir and 32 injections of nivolumab during 1.3‐year period. Both therapies are still ongoing. The progression of disease and therapy dates is represented in Figure 6.

At 9.8 months, no lung nodules or enlarged lymph nodes in mediastinum were detected (Figure 2B). Lymph node in left mesentery had decreased in size to 1 cm (Figure 4B). Lesion in right liver lobe had disappeared and lymph node in hepatic hilus decreased in size (Figure 5C,D).

After 1.2 years, the disease progressed to lymph nodes in the mesogastrium (Figure 4C); however, the lung metastases were stable (Figure 2C). The patient underwent laparoscopic resection of a mesogastric lymph node at 1.5 years; the histopathological report after surgery verified melanoma metastases in one of six lymph nodes in the mesentery of the small intestine. Tumor cells exhibited severe nuclear atypia, 8 mitosis in 10 HPF (approx. 1.5 mitoses per 1 mm^2^) and necrosis of about 50% of tumor area. No extranodal spread was visible. PD‐L1 immunohistochemical reaction (with antibodies of Dako clone 22C3) was performed on a posttherapy metastasis and a primary skin melanoma sample; the reaction was negative in both samples (weak incomplete membranous reaction was observed in less than 1% of the melanoma cells).

At 1.5 years, the CT scan suggests that the lesion in left mesentery has been reduced in size (Figure 4D). No lesions were observed in liver, hepatic hilus, or spleen (Figure 5E,F). Overall, the radiological findings are stable in comparison with findings at 1.3 years (Figure 3J,K,L, 4D, 5G).

No other health problems have been reported. Current ECOG performance status is 0.

Throughout the treatment with Rigvir, the patient has not reported any side effects; also, tolerance of nivolumab has been satisfactory. The patient noted some neuropathic pain after nivolumab infusions. After the patient received the first nivolumab injection (before start of Rigvir treatment), there was a slight elevation of blood glucose that remains slightly elevated.

It has previously been reported that nivolumab may cause impaired glucose tolerance or autoimmune diabetes.^8^, ^9^, ^10^, ^11^ The available blood sample tests from 6.6 months to 1.3 years show that all values, except for glucose values (Grade 1 according to NCI CTCAE), were in the normal range. At 7.6 months, the overnight fasting blood glucose was reported to be 6.1 mmol/L, at 1.1‐year 6.2 mmol/L and at 1.3‐year 6.1 mmol/L. A slight elevation of blood glucose levels has been observed both in melanoma patients that are only observed and that have been treated with Rigvir.^7^

The patient was a long‐term smoker (20 cigarettes per day) for about 18 years; smoking was discontinued 2‐3 months after diagnosis. The patient has worked as a civil engineer for many years and has had high sun exposure, working outdoors without the protection from sun.

## 3 DISCUSSION

Dendritic cells take up fragments of dying cancer cells and display them as tumor‐associated antigens.^12^ Dendritic cells migrate to tumor‐draining lymph nodes where T cells are primed. Activated T cells migrate back to the tumor, recognize, and lyse tumor cells. However, tumors have mechanisms that help them to escape the immune system. Tumor cells may prevent dendritic cell maturation; then T cells are not activated, and immune tolerance is induced. Inhibited dendritic cell maturation also has a negative effect on production of chemokines, which normally recruit T cells to the tumor. Tumors also affect the vascular endothelium. Factors like vascular endothelial growth factor suppress production of adhesion molecules required for T‐cell adhesion to endothelium and induce expression of immune inhibitory and cytotoxic molecules.^12^

Only a limited number of patients respond to PD‐1 blockade therapy. The explanation appears to be that if no CD8^+^ T cells are present in the tumor, the patient will not respond to the therapy. Therefore, it is important to attract CD8^+^ cells into the tumor microenvironment, which would improve the effect of PD‐1 antibodies.^1^ Oncolytic viruses have been demonstrated to besides tumor lysis, also activate antitumor immune response.^13^

Several recent clinical trials have investigated the possible synergistic effect of immune checkpoint inhibitors with oncolytic viruses. For example, in a phase Ib study, Coxsackie A21 virus was administered in combination with pembrolizumab and showed preliminary best overall response (complete and partial response according to immune‐related response criteria) rate in 61.0% (14/23 patients). Combination of Coxsackie A21 and pembrolizumab showed clinical benefit, and the possible synergistic mechanism was explained by increased immune cell infiltration into tumor lesions, activation of RIG‐I pathway, upregulation of gamma‐INF response and key immune checkpoint genes, including PD‐L1, caused by intratumoral Coxsackie A21 virus injections. Upregulation of PD‐1L expression on tumor cells increases the effect of pembrolizumab.^14^

Combination of talimogene laherparepvec and ipilimumab has been evaluated in a phase Ib and phase II studies, both showing higher efficacy in combination than as a monotherapy of talimogene laherparepvec or ipilimumab alone.^15^, ^16^

Talimogene laherparepvec intralesional injections before pembrolizumab administration promoted the CD8^+^ cell infiltration into tumor. Systemic increase in CD4^+^ and CD8^+^ cells was also observed. CD8^+^ T cells express PD‐1 receptor that binds to tumor PD‐1L; therefore, increased CD8^+^ cell count could benefit to combination therapy with anti‐PD‐1 antibodies.

A case series evaluated talimogene laherparepvec in combination with PD‐1‐based immunotherapy in unresectable stage III and IV melanoma patients. Ten patients with unresectable stage IIIC to IVM1b melanoma were treated with talimogene laherparepvec and checkpoint inhibitor. All patients had different treatment plans as well as previous treatment history. The study was too short to provide median survival or 12‐month survival rates; however, complete response was observed in 60% of the patients.

The data suggest that radiation therapy prior to immune therapy may enhance the effect of the immune therapy. Radiation therapy induces the release of tumor‐associated antigens that, in turn, play essential role in priming adaptive immune system^17^, ^18^; some encouraging reports have been published.^19^, ^20^ However, the present patient experienced disease progression after the radiation therapy.

Overall, the present result suggests that combination of an oncolytic virus and an immune checkpoint inhibitor could be of benefit for patients without causing additional toxicities. The present clinical case shows a patient that had a rapidly progressing metastatic melanoma. The combination of oncolytic virotherapy with Rigvir and a PD‐1 antibody has slowed the progression. The combination is well tolerated since no new toxicities have been reported.

## CONFLICT OF INTEREST

LB, IJ, AT, AR and PA are employees of the AmberLife cancer clinic and the Rigvir, respectively; EO and DP have no disclosures.

## AUTHORS' CONTRIBUTIONS

LB, IJ, AT, EO, AR, and PA: made substantial contributions to acquisition of data, analysis, and interpretation of data and drafted manuscript. EO and DP: carried out the radiology and histology expert analysis, respectively. LB: was the attending oncologist of the patient. IJ: was the attending immunologist of the patient. All authors have read and approved the final version for publication.

## CONSENT FOR PUBLICATION

Written consent to publication has been obtained from the patient.

## Data Availability

All relevant data and materials are included in the manuscript.
